# Abstract “why” Thoughts About Success Lead to Greater Positive Generalization in Sport Participants

**DOI:** 10.3389/fpsyg.2015.01783

**Published:** 2015-11-23

**Authors:** Jens Van Lier, Michelle L. Moulds, Filip Raes

**Affiliations:** ^1^University of Leuven, Leuven, Belgium; ^2^University of New South Wales, Sydney, NSW, Australia

**Keywords:** sport psychology, retrospective thinking, cognitive processes, positive generalization, self-esteem

## Abstract

Generalizing from a single failure or success to future performances and their self-concept could have an important impact on sport participants. This study examined the impact of the way sport participants think about success on positive generalization. Sport participants (*N* = 222) completed an online experimental study in which they were induced to think about meanings, causes and implications (i.e., abstract-“why”-thinking) or about more perceptual concrete aspects of their performance (i.e., concrete-“how”-thinking). We hypothesized that abstract-“why”-thinking would lead to greater positive generalization and that this effect would be moderated by self-esteem. Our results supported our hypothesis that abstract thinking increased positive generalization, and this effect was more clearly visible in sport participants with higher self-esteem. These results suggest that retrospective thinking about the “why” of a good performance may benefit athletes in the long run because they generalize the outcome to future performances and their self-concept which may boost their motivation and consequently their performance.

## Introduction

The way in which one thinks about or dwells on past failures can have good or bad implications for self-esteem, motivation, future performances, and other aspects of the self. Indeed, (repetitive) thought processes (e.g., rumination, worry, counterfactual thinking, etc.) that are focused on negatively valenced content can have both constructive and unconstructive consequences (Watkins, 2008). One aspect of repetitive thought about negative content that has been consistently found to be unconstructive is thinking about “why” (i.e., adopting an abstract processing style) rather than thinking about “how” (i.e., adopting a concrete processing style; Watkins, 2008). However, less is known about the implications of “why” and “how” repetitive thinking that is focused on positive content such as success performances. Moreover, such post-event retrospective cognitions and their consequences are highly understudied in the context of sports (Uphill and Dray, 2009).

The way in which athletes respond to a single defeat or victory might have crucial implications for their next training session, their next game/race, a more prolonged period in their upcoming season or even consequences for their sense of self-worth. In other words, athletes could generalize a single failure or success performance to future performances or even to the totality of their self-concept. For example, a cyclist who was not able to stay with the first group on a climb in the first race of the season (single failure) might believe that he/she will not be able to follow the next race on a different, more flat, course. Moreover, he/she may start to believe that the whole season will not work out at all and that they are a complete failure. Conversely, another cyclist who was able to just hang on to the first group on that climb, which he/she considered to be a success, might believe that they will be able to follow the next race on a different, more flat, course. Moreover, they may start to believe that this whole season will work out just fine, which in turn might boost their confidence that they are good cyclists. In these ways, both failure and success generalizations may have a significant impact on these cyclists’ motivation for the season ahead.

Negative (over)generalization is a central concept in the depression literature where it is commonly defined as “unjustified generalization on the basis of a single incident” (Beck, 1976, p. 94). In essence, generalization is actually adaptive because it helps people to transfer knowledge over situations (Hermans et al., 2013). However, negative overgeneralization, as seen in depression and individuals with low self-esteem (e.g., Kernis et al., 1989; Carver, 1998; Libby et al., 2011), refers to generalization that is inappropriate because it is excessive (Epstein, 1992). The vast majority of the (over)generalization literature consists of studies on negative generalization (i.e., generalization following negative events, failures; e.g., Kernis et al., 1989; Carver, 1998; Fulford et al., 2012). Moreover, in two experimental studies with negative stimuli, it has been shown that abstract processing (relative to concrete processing) increases negative generalization (Van Lier et al., 2014, 2015). The present study examines the impact of processing styles on positive generalization (i.e., generalization following positive events such as a good performance).

Following a negative event, abstract processing has been found to have maladaptive consequences. Experimental manipulations of abstract (vs. concrete) processing styles have produced negative outcomes (in addition to increased negative generalization), such as poor problem solving (Watkins and Moulds, 2005), poor emotional recovery after failure (Watkins, 2004), the retrieval of overgeneral autobiographical memories (Raes et al., 2008), negative global self-judgments (Rimes and Watkins, 2005), and feelings of worthlessness and competency (Vassilopoulos and Watkins, 2009). In the context of sports, Maxwell (2004) found that anger rumination (although not specified whether this was abstract or concrete rumination) predicted subsequent athlete aggression.

An abstract processing style is a thought process that is characterized by “general, superordinate, and decontextualized mental representations that convey the essential gist and meaning of events and actions, such as representations of “why” an action is performed and of its ends and consequences” (Watkins, 2008, p. 187). Hence, when thinking abstractly, individuals think about the causes, meanings and implications of actions or events (Moberly and Watkins, 2006). In contrast, a concrete processing style is a thought process that is characterized by more “low-level mental representations that include subordinate, contextual, and incidental details of events and actions, such as representations of the specific “how” details of an action and of the means to an end” (Watkins, 2008, p. 187). Thus, when thinking concretely, individuals think in a more moment-to-moment-, more perceptually oriented, way. For example, concrete thought focuses on how the event unfolded or what one could hear, smell and touch during the event (Moberly and Watkins, 2006).

Note that causal attributions (e.g., Biddle et al., 2001) may be a crucial part of abstract processing an event. Causal attributions have indeed been shown to influence future beliefs about success (e.g., Le Foll et al., 2008). More specifically, Weiner (1986) argued that the stability of the attribution is important for future expectancies of success. Hence, after failure it would be functional to see a cause as unstable, whereas following success it would be functional to see the cause as a stable factor (Le Foll et al., 2008). Moreover, other research (e.g., Grove and Pargman, 1986) suggests that it is the controllability of the attribution that is important. In short, research has shown that the controllability, the stability of the attribution and also seeing attributions as internal may have a crucial impact on future expectancies (Le Foll et al., 2008). However, while abstract and concrete thought is more at the level of the “process” aspect of thought (See Watkins, 2008, p. 185), attributions are more about “content” aspects. Hence, in this study, we examined thoughts at the process level; specifically, we investigated the implications of the level of construal (abstract vs. concrete processing; Trope and Liberman, 2003) that individuals adopted while thinking about success. When individuals process events they may do so in an abstract way (i.e., high level of construal; Trope and Liberman, 2003) or a concrete way (i.e., low level of construal, Trope and Liberman, 2003).

In contrast to the maladaptive effects of abstract processing following negative events or failure, the effects of abstract (vs. concrete) processing following positive events has, however, not been extensively tested (but see for example, Hetherington and Moulds, 2013). Watkins (2008) posited that the mechanism by which abstract processing could have an unconstructive consequence might be via its impact on the degree of generalization in response to negative emotional events (Van Lier et al., 2014, 2015). In contrast, Watkins (2011) hypothesizes that abstract processing of positive events may have constructive consequences while concrete processing of positive events may have neutral to maladaptive consequences. Hence, related to the effect of abstract processing on negative generalization, it may be the case that abstract processing could have constructive consequences following positive events because this type of thinking promotes positive generalization.

With regard to the effects of abstract processing of positive events, Marigold et al. (2007) showed that when participants who were low in self-esteem were asked to abstractly describe a compliment from their partner, they felt better about the compliments, about themselves and their relationships. Hetherington and Moulds (2013), however, found no effect on subsequent positive affect when participants were induced in either an abstract or concrete processing style during a success task. In the same vein, fewer studies have been examining positive generalization (i.e., generalization following positive events, successes; but see Klar et al., 1997; Eisner et al., 2008). Therefore, this study set out to examine the impact of styles of post-event retrospective processing, in the form of abstract “why” versus concrete “how” thoughts, on positive generalization (i.e., following a success performance). In the same vein of Watkins (2008), we hypothesized that abstract processing following success would have constructive consequences insomuch as it would increase positive generalization, as compared to a concrete processing style.

In the present study, recreational sport participants involved in different competitive sports were asked to describe their latest competitive event. The participants also rated how well they thought they had performed. Then, participants were prompted to think back on this performance in either an abstract (e.g., “What did your performance mean to you?”) or a concrete way (e.g., “Play out the performance in your mind. What could you see around you? What did you see?”). In order to assess positive generalization over the future, participants were asked to make predictions about the probability of them performing well in the future (e.g., “Next game/race”). Additionally, in order to assess positive generalization to their self-concept, participants also completed a generalization from success questionnaire.

We hypothesized that participants who were instructed to think back about their good performance adopting an abstract processing style (relative to a concrete processing style) would indicate a higher probability of a good performance in the future and would score more highly on the generalization from success questionnaire. If these predictions are supported, the findings would indicate that an abstract processing style has an adaptive impact following success and in turn, increases positive generalization.

It has been posited that in general, abstract processing of positive situations is adaptive but might become maladaptive in the context of dysphoria (Watkins, 2008, 2011). Moreover, with regard to failures, individuals with low self-esteem tend to have more negative reactions compared to individuals with high self-esteem (e.g., Kernis et al., 1989; Libby et al., 2011). Likewise, we theorized that self-esteem might also act as a crucial factor following a good performance. Therefore, we hypothesized that individuals with low self-esteem (analogous to people with dysphoria) would not benefit from either concrete or abstract processing of their good performance, whereas individuals with high self-esteem would benefit more from an abstract processing style (see for a similar hypothesis for dysphoric participants Hetherington and Moulds, 2013). One possible reason as to why individuals with low self-esteem may not benefit from an abstract processing style might be that abstract processing would potentially open the door to dampening cognitions: “Maybe I don’t deserve this success,” “Maybe I was just lucky” etc. (e.g., Wood et al., 2003)^1^.

## Materials and Methods

### Participants

Participants were recruited from the online study platform MTurk (Amazon’s Mechanical Turk) with the restriction that they needed to be U.S. residents and have a 95% task approval rate for their previous HITs (Human Intelligence Tasks). A link to a short qualification survey was posted on MTurk. This qualification survey consisted of demographics (Age, Nationality, Country of Residence, First language, Gender, Ethnicity, Marital status, Education, Employment) and 2 other questions. It was crucial that participants responded “yes” to the first question “Do you play or do any sport in competition (recreational, amateur, professional, etc.)?” in order to qualify as eligible to take part in the study. The second question “Have you ever donated blood?” was actually irrelevant to the study. All of the abovementioned questions were included in order to suggest that certain demographics or other variables may be a pre-requisite to qualify to take part in the study, but we did not specify the answers that would disqualify a potential participant.

Individuals did not qualify when they were below the age of 18 or when they answered “No” to the sports-question. These individuals received the message “Unfortunately you do not meet the study criteria, we thank you for your interest.” Individuals who did qualify received the message that they met the selection criteria for the study and received a qualification code that they needed to copy and enter at the beginning of the study to complete the HIT. In total 260 participants completed the HIT. The participants were paid $3. Participants provided their consent to take part in a 30-min study on thinking styles about competitive sports events in which they would be asked to complete multiple questionnaires. The experiment in this study was conducted in accordance with the Declaration of Helsinki and approved by the Human Research Ethics Advisory (HREA) Panel C—Behavioral at The University of New South Wales.

### Materials and Methods

#### Sport-related Questions

In this section participants had to complete sport specific questions such as: “Do you play or do any sport in competition?”; “Do you compete in an individual or team sport?”; “Which sport do you compete in?”; “On which level do you compete?”; “on average, how many times a week do you engage in your sport-related activity?”; “How many hours do you spend (training and game) per week on average during your sport season?”

#### Rosenberg Self-esteem Scale (Rosenberg, 1965)

Participants completed the 10-item RSES with a scale ranging from 0 (*strongly agree*) to 3 (*strongly disagree*). This scale is a popular and well-validated measure of global self-esteem. Internal consistency for the RSES in this sample was good (α = 0.93).

#### Attention Check Question

Mixed in-between the items of the self esteem scale there was one item reading: “I am not reading the questions of this survey.” This item served as the attention check question.

#### Attitudes Toward Self—Generalization (Carver and Ganellen, 1983)

The participants completed the generalization subscale of the ATS. This subscale consisted of four items with a scale ranging from 1 (*I agree a lot*) to 5 (*I disagree a lot*) and assesses the tendency to generalize from a single failure to the broader sense of self-worth. Internal consistency for the generalization subscale of the ATS in this sample was good (α = 0.84).

#### Positive Over-generalization (Eisner et al., 2008; Carver and Johnson, 2009)

The POG is a 16-item self-report questionnaire that measures the generalization from a positive outcome to the respondent’s broader sense of self. The POG has three subscales: “lateral generalization” (i.e., that is, from a good outcome in one domain to positive outcomes in other areas of life or life in general; Eisner et al., 2008, p. 159), “upward generalization” (i.e., from one good outcome to a more expansive outcome in the same general domain; Eisner et al., 2008, p. 159) and “social generalization.” The POG in this sample had a good internal consistency (α = 0.90).

#### The Action Control Scale—Preoccupation Subscale (ACS-90, Kuhl, 1994)

The ACS-90 consists of 12 scenarios (e.g., “When I am in a competition and have lost every time”). For each scenario, participants are asked to indicate whether they would engage in either a ruminative response (e.g., “The thought that I lost keeps running through my mind,” scored 1) or a non-ruminative response (e.g., “I can soon put losing out of my mind,” scored 0). The scale has good reliability and validity (Kuhl, 1994). Internal consistency for the ACS-90 in this sample was good (α = 0.87).

#### Repetitive Thinking Scale-trait (Samtani and Moulds, unpublished)

The RTS-T is a 24-item self-report questionnaire that measures abstract and concrete processing of stressful and challenging situations. Hence, the RTS-T has an abstract and concrete processing subscale. Each item is scored on 5-point scale ranging from 0 (*never*) to 4 (*almost always*). Internal consistency for the RTS-T in this sample was good (α = 0.95).

#### Depression Anxiety Stress scale (DASS 21)

The DASS 21 is a 21-item self-report questionnaire that measures negative emotional states of depression, anxiety and stress (Lovibond and Lovibond, 1995). Each item is scored on 4-point scale ranging from 0 (“*did not apply to me at all*”) to 3 (“*applied to me very much, or most of the time*”). In line with the manual for the DASS 21, we multiplied the total values of the DASS 21 scales by two (Lovibond and Lovibond, 1995). Internal consistency for the DASS 21 in this sample was good (α = 0.95).

#### Positive and Negative Affect Scales (Watson et al., 1988)

The PANAS is a 20-item self-report questionnaire that consists of two 10-item scales. The two scales are measuring positive affect (e.g., “interested,” “excited”) and negative affect (e.g., “distressed,” “upset”). Participants are asked to rate on a 5-point scale the extent to which each item reflects how they feel at that point in time with a scale ranging from 1 (“*very slightly*”) to 5 (“*extremely*”). The PANAS is a reliable and valid measure of affect (Watson et al., 1988). Internal consistencies for the pre- and post-PANAS in this sample were good (α = 0.87 and α = 0.88).

#### Questions About Latest Sport Performance

Participants were asked to think back to their latest competitive performance (race, game, etc.) and write down in a couple of sentences which race, game, etc. it was. Following this description participants were asked to indicate how long ago this competitive performance was (i.e., ranging from “*one day ago*” to “*more than a month ago*”); the outcome (i.e., win, lost, first 10, podium, personal best, etc), and importantly, to provide their subjective rating of how well they thought they performed in their race/game. This last question was anchored on a 7-point scale ranging from “*very bad*” to “*very good*.”

#### Abstract/concrete Induction

Participants were asked to think about their performance in the game/race. The inductions of abstract and concrete processing styles were modeled on that used by Moberly and Watkins (2006). In the abstract condition participants were instructed to think about why they performed the way they did. “Think about the meanings and consequences of your performance. Think about what this performance says about you as a person. Think about this performance in words and meanings, by using verbal language, as if you were talking.” In the concrete condition participants were instructed to focus on the game/race. “Let your performance in that game/race play out again in your mind, just like you play a video about the performance; make a detailed picture about your performance at the game/race in your mind.”

Following these instructions participants were instructed to answer every question on the next page at their own pace in order to further reinforce the induced processing style. They were asked to write a minimum of a half a line and a maximum of three lines per question, and to use full sentences to answer the questions. The abstract questions were: “What did your performance mean to you?”; “What were the consequences and implications of your performance for you?”; “How did you think about yourself after your performance?”; “Why did you feel the way you felt after your performance?”; “Why did you perform the way you did?”; “What do you think about your performance? What does this performance say about your capacities?”; “Was your performance like you had expected? Why was it or why was it not like you expected?”

The concrete questions were: “Play out the performance in your mind. What could you see around you? What did you see?”; “Play out the performance in your mind. What could you smell? Was the air fresh? Was it cold/warm?”; “Play out the performance in your mind. What could you hear?”; “Play out the performance in your mind. Which feelings occurred during your performance?”; “Play out the performance in your mind. What were the physical sensations you felt during your performance?”; “Play out the performance in your mind. What did you do right before the game/race?”; “Play out the performance in your mind. What did you do after the race/game or the rest of the day?”

#### Positive Generalization About the Future

To assess participants’ ratings of the probability of a good performance in the future, participants were asked to imagine themselves in the future and in their future performances. Next, they were asked to indicate the likelihood that their future performance would be the same as the performance that they described in this study. That is, they were asked how likely it is that they will perform this way again in the future. They were instructed to not think too much about it, but to clearly represent their opinion. Participants could indicate their likelihood estimation by ticking on a horizontal axis with anchor points of 0 (“*I will certainly NOT perform like this*”) to 100 (“*I will definitely perform like this*”). The situations in the future for which they were asked to give their likelihood estimation were: “Next training session?”; “Next game/race/competitive event?”; “Next month?”; “Whole Season?”; “Next season?”; “Whole career?” Internal consistency for this scale in this sample was good (α = 0.94).

#### Attitudes Toward Self—Generalization Particular Event (Negative and Positive)

This questionnaire is an adapted version of the original ATS-generalization (Carver and Ganellen, 1983) in the sense that this version measured the extent to which respondents generalized from their particular identified competitive event, rather than measuring a trait-like tendency to generalize (i.e., the original ATS). We used the rewording to a particular event employed by Libby et al. (2011). As such, respondents were asked to rate how much they agreed or disagreed with the following statements on a scale from 1 (“*I agree a lot*”) to 5 (“*I disagree a lot*”): “When I think about this performance, I feel like I am a failure”; “Even though this performance is a failure, it’s just a one-time occurrence where I did not meet a specific goal” (reverse-scored); “When I think about this performance, I wonder if I can do well at anything at all”; “This single performance influences how I feel about myself overall.”

However, our study examined the generalization of success. Therefore, we adapted this scale to measure generalization of success instead of failure. Specifically, the items were re-worded as follows: “When I think about this performance, I feel like I am a success”; “Even though this performance is a success, it’s just a one-time occurrence where I met a specific goal” (reverse-scored); “When I think about this performance, I feel if I can do well at everything”; “This single performance influences how I feel about myself overall.” Internal consistency for this scale in this sample was acceptable (α = 0.64).

### Procedure

The study was posted on the MTurk platform as a study on thinking styles about competitive sports events that consisted of completing questionnaires. We indicated that the study would take approximately 30 min to complete. If participants qualified as eligible to take part, (see participants section for details) they received a qualification code to enter at the start of the actual study. The participants gave consent by ticking the box under “I give consent” on the screen. Participants were randomly allocated to either the abstract or concrete condition. All participants went through the same questionnaires in an identical order before they received an abstract or concrete processing induction (i.e., the abstract and concrete questions about their selected performance).

The order of questionnaires and induction was as follows: “Demographics and sports-related questions”; “RSE”; “ATS-generalization”; “RTS-T”; “DASS 21”; “POG”; “ACS-90”; “PANAS (pre-induction)”; “Questions about latest sport performance”; “Abstract/concrete induction”; “Generalization in their sport”; “Generalization about self in domains other than sport”; “Attitudes Toward Self—generalization particular event” and “PANAS (post-induction).” Participants who rated their performance with a 5 (“*Fair*”) or above they received the positive ATS- generalization particular event whereas when they rated their performance with a 3 (“*Poor*”) they received the negative ATS- generalization particular event. Following the completion of all questionnaires the participants had the opportunity to write any comments or inform us about any technical issues they had completing the study. Finally, participants were guided to the debriefing page where they received information about the study and further contact details.

### Data Analysis

Independent *t*-tests were used on the control variables to check for differences between individuals in the abstract condition vs. individuals in the concrete condition. In order to ensure that the amount of words written and the change in mood was equal across experimental inductions, an independent *t*-test on the amount of words and a repeated measures ANOVA on mood pre- and post-experimental induction was conducted. Our main hypothesis was analyzed by a linear regression on positive generalization over the future (total amount of generalization averaged over the 6 items) and generalization to the self-concept (total score on 4 items) with condition as a between-subject factor (abstract vs. concrete processing) and self-esteem as a continuous predictor in our model, whilst controlling for chronic tendency to generalize. For all analyses, the alpha level was set at *p* < 0.05.

## Results

### Participant Characteristics

Out of the 260 individuals that completed the full study, 20 individuals rated their performance as negative (3 or below), 14 as neutral and 226 as positive (5 or above). Our sample size to assess negative generalization was too small and therefore those participants as well as the participants with a neutral rating of their performance were excluded from the further analyses. Furthermore, four participants out of the 226 failed the attention check question and were therefore also excluded. Hence, a total of 222 individuals (142 males) were included in the study. Their mean age was 32.93 years (SD = 10.68; age range: 18–69). Table 1 displays the frequencies, means and standard deviations of the demographics, sports questions and the questionnaires.

**TABLE 1:** **Frequencies, means and standard deviations of study measures**.

	**Percentages**	**Abstract *M* (SD)**	**Concrete *M* (SD)**
***Demographics***			
Age		33.01 (11.21)	32.86 (10.20)
Gender (%Female)	36%		
English 1st Language	98.6%		
White/Caucasian	80.6%		
Black/African American	11.3%		
Other	8.1%		
High school	35.1%		
Bachelor’s degree	56.3%		
Master’s degree or advanced	7.7%		
***Sports***			
Professional	1.4%		
College/University	5.4%		
Amateur	25.7%		
Recreational	67.6%		
***Study questionnaires***			
RSES		23 (5.20)	22.27 (5.63)
DASS depression		5.54 (8.75)	6.27 (8.00)
DASS Anxiety		6.04 (8.43)	5.96 (7.21)
DASS stress		8.29 (8.51)	10.12 (8.26)
ATS generalization^†^		8.74 (3.89)	9.72 (3.81)
RTS Abstract		27.33 (7.61)	27.94 (7.65)
RTS concrete		31.12 (8.91)	31.15 (8.41)
POG lateral		22.83 (4.90)	23.75 (3.63)
POG upward		11.99 (4.76)	12.75 (4.95)
POG social		11.39 (4.65)	12.10 (4.71)
ACS-90 pre-occupation	4.98 (4.10)	5.62 (3.49)	
Generalization future	72.19 (23.56)	66.24 (19.98)	
Generalization self	14.31 (3.14)	13.70 (2.93)	

^†^p < 0.07; DASS, Depression and Anxiety Stress Scale; RSES, Rosenberg Self-Esteem Scale; ATS, Attitudes Toward Self; RTS, Repetitive Thinking Scale; POG, Positive Overgeneralization; ACS, Action Control Scale.

Out of the 222 included participants, 68 (30.6%) participants rated their sports performance as “fair,” 119 (53.6%) participants as “good” and 35 (15.8%) participants as “very good.” The abstract and concrete group only marginally differed on the ATS-generalization, *t*(220) = –1.88, *p* = 0.06. For 166 participants (74.8%) their latest competitive event occurred in the last week or more recently. Chi-Square tests indicated that the proportion of participants that had their latest performance 1 week or less than a week ago did not differ between processing style conditions (*p* = 0.64).

### Induction

The amount of characters that were written as answers to the questions for the induction of abstract or concrete processing did not differ between the abstract and concrete induction, *t* = –1.45 (abstract *M* = 94, SD = 42; concrete *M* = 103, SD = 46).

### Mood

To check whether the inductions of abstract and concrete processing modes had a differential effect on mood, a 2 (Condition: abstract vs. concrete) × 2 (Time: pre- vs. post-induction) repeated measures ANOVA was conducted with negative and positive affect as dependent variables. For negative affect, there was no main effect of Time, *F* < 1, and a marginally significant Condition × Time interaction, *F*(1,218) = 2.93, *p* = 0.09, ${\text{η}}_{\text{p}}^{\text{2}}$ = 0.01. For positive affect, there was a main effect of Time, *F*(1,218) = 15.88, *p* < 0.001, ${\text{η}}_{\text{p}}^{\text{2}}$ = 0.07, and a Condition × Time interaction, *F*(1,218) = 8.28, *p* < 0.01, ${\text{η}}_{\text{p}}^{\text{2}}$ = 0.04. Paired samples *t*-tests revealed that participants in the concrete condition did not change in mood, *t* < 1, whereas participants in the abstract condition increased in positive affect from pre- to post-induction, *t*(107) = –5.94, *p* < 0.001.

However, when we compare mood at pre- and post-induction there were no differences between the abstract and concrete condition, *t* < 1, and *t* = 1.06, respectively.

In our predictions about generalization we made specific claims that the effect of the induction would interact with self-esteem. Therefore, in this section it seems warranted that we also look at this specific interaction. However, besides a main effect of self-esteem, there were no significant effects.

### Dependent Variables

We predicted that participants who thought about their performance in an abstract way (“Why,” “Reasons,” “Causes”) compared to a concrete way (“How,” “Perceptual”) would show greater overgeneralization from the particular sports performance. We also predicted that this effect would be moderated by self-esteem. We ran a linear regression analysis for each dependent variable separately. The model included self-esteem (centered around its mean), condition (abstract vs. concrete), and their interaction. Moreover, we also controlled for chronic tendency for positive generalization (i.e., “lateral generalization” POG centered around its mean) and its interaction with condition. That way, we were able to isolate the relationship between processing mode about the recalled sport performance and generalization from that particular performance.

#### Positive Generalization Over the Future

For generalization in their sport, we found an effect of Condition, *t*(214) = –2.18, *p* < 0.05, β = –0.14, an effect of Self-Esteem, *t*(214) = 3.48, *p* < 0.01, β = 0.34, and a marginally significant Condition × Self-Esteem interaction, *t*(214) = –1.95, *p* = 0.05, β = –0.19 (Table 2). As predicted, participants in the abstract condition showed more positive generalization relative to participants in the concrete condition. The interaction suggests that this effect is larger for participants with higher self-esteem^2^.

**TABLE 2:** **Results of the regression analysis for positive generalization over the future and their self-concept**.

**Variables**	**B**	**SE**	**β**	***t***	***p***
***Positive generalization over the future***					
Condition^1^	–6.18	2.83	–0.14	–2.18	0.03
Self-esteem	1.38	0.40	0.34	3.48	0.001
Condition × Self-esteem	–1.06	0.54	–0.19	–1.95	0.05
Lateral positive generalization	0.63	0.43	0.12	1.46	0.15
Condition × Lateral Positive Generalization	0.71	0.72	0.08	0.99	0.33
***Positive generalization to their self-concept***					
Condition^1^	–0.76	0.36	–0.13	–2.11	0.04
Self-esteem	0.19	0.05	0.34	3.79	<0.001
Condition × Self-esteem	–0.13	0.07	–0.17	–1.91	0.06
Lateral positive generalization	0.25	0.06	0.35	4.61	<0.001
Condition × Lateral Positive Generalization	0.07	0.09	0.06	0.78	0.44

^1^Condition was dummy coded with abstract = 0 and concrete = 1

#### Positive Generalization to Their Self-concept

For generalization to the self, we found an effect of Condition, *t*(214) = –2.11, *p* < 0.05, β = –0.13, an effect of Self-Esteem, *t*(214) = 3.79, *p* < 0.001, β = 0.34, and a marginally significant Condition × Self-Esteem interaction, *t*(214) = –1.91, *p* = 0.06, β = –0.17 (Table 2). As predicted, participants in the abstract condition showed more positive generalization relative to participants in the concrete condition. The interaction suggests that this effect is larger for participants with higher self-esteem^3^.

## Discussion

The present study sought to examine the effect of an abstract (“Why”) versus a concrete (“How”) way of thinking back about a good performance on recreational sport participants’ generalization of this success to the future and to their self-concept. We hypothesized that participants in the abstract processing style condition (relative to participants in the concrete processing style condition) would show higher probability judgments of success in the future and engage in more positive generalization. Such a finding would show that abstract processing of a positive event (e.g., a good performance) is actually adaptive (see Watkins, 2011). We expected that the adaptive effect of an abstract processing style would be moderated by self-esteem. However, the literature has found contradictory results with regard to the direction of this relationship.

As hypothesized, abstract processing about a positive event (i.e., a good performance) increased positive generalization relative to a concrete processing style. In fact, two different aspects of positive generalization were measured, namely generalization about future events following a single event (e.g., Klar et al., 1997) and generalization to the self-concept (e.g., Carver and Ganellen, 1983). The results of the former measure showed that an abstract processing style about a single good performance increased positive generalization about future events (e.g., next game/race) and the results of the latter measure showed that an abstract processing style about a single good performance increased positive generalization to the self-concept.

The hypothesis that the effect of processing style would be moderated by self-esteem could not be clearly confirmed in this study. That said, our results suggest that the effect of abstract thinking is more clearly present in individuals with high levels of self-esteem. This sample consisted of individuals with fairly normal to high self-esteem (Schmitt and Allik, 2005) and, therefore, caution should be applied when generalizing these findings to individuals with low self-esteem or dysphoria. It could nevertheless be possible that individuals with low self-esteem actually show maladaptive consequences as a result of processing positive events abstractly. For example, when thinking about the causes and implications of a good performance, athletes with low self-esteem might actually “dampen” this positive event (e.g., “I did not deserve this win”; Wood et al., 2003; Feldman et al., 2008). Abstract processing in low self-esteem individuals may potentially open the door to dampening cognitions: “Maybe I don’t deserve this success,” “Maybe I was just lucky” “You’ll see, next time things won’t go that smoothly” etc. Additionally, for individuals with low self-esteem, attributions about the causes of their good performance (e.g., controllability and/or stability; Coffee and Rees, 2008; Coffee et al., 2009) may well prove to be critical.

In the present study, however, abstract thinking (“Why”) about a good performance may have in general enhanced more global, stable and/or more universal attributions (Coffee and Rees, 2008), whereas concrete processing (“how”) may have actually decreased the chance for any such attributions to be made. These more global, stable and/or more universal attributions may be one of the reasons that abstract processing of a good performance led to greater positive generalization. One interesting avenue for future research could be to investigate the nature of the attributions that athletes generate as a result of thinking about the causes, meanings and implications of a good performance (i.e., abstract thinking).

For a negative event, such as a poor game/race, it is plausible that people with low self-esteem show more negative overgeneralization. Consistent with this hypothesis, Brown and Dutton (1995) found that people with low self-esteem showed more severe emotional reactions (e.g., pride, humiliation) following a failure because they overgeneralized the implications of this failure. Hence, in recreational sport participants and athletes with low self-esteem a concrete processing might be more adaptive following failure because this processing style would diminish its overgeneralization (see for the effects of processing style on generalization in negative situations: Van Lier et al., 2014). Future research could test this hypothesis in athletes following a failure performance. Another future avenue for this research area would be to examine the influence of having a coach or using performance technologies (e.g., heart rate monitor) on the abstract/concrete thoughts of sport participants and hence the impact of those variables on individuals’ generalization.

This study has several limitations. First, this study was conducted online via the online platform MTurk. As such, we did not have direct control on the environment in which the participants took part in the experiment. However, other studies indicate that participants with a 95% task approval rate display reliable and high quality data (e.g., Buhrmester et al., 2011; Peer et al., 2013). Second, this study consisted of athletes that were recruited from the community. Hence, the sample consisted mostly of recreational and amateur sport participants, and professional athletes were underrepresented in our sample. Therefore, future studies could specifically recruit professional athletes, as it might be possible that more expertise in their sport may result in different reactions to abstract and concrete processing styles. Indeed, it may be the case that the effects that we observed may be even larger in professional athletes because they invest more time in their sport, and furthermore, because their sport could arguably be more important for their self concept. Therefore, the implications of their processing style on positive generalization might be enhanced for these individuals. Third, future studies could include a measure of the trait tendency to use imagery (e.g., Spontaneous Use of Imagery Scale; Reisberg et al., 2003) in order to check that there were no between-condition differences on this variable. Another limitation of the current study is that we do not have a control condition. We cannot make strong claims as to whether the observed effects are caused by the active increasing effect of abstract processing or the active diminishing effect of concrete processing on positive generalization. Also, some questionnaires at the beginning stage were not necessary for the purpose of the study and therefore too many questions might have increased impatience of participants and resulted in less accuracy of their answers. However, any such effects would be uniform across participants as all participants completed an identical battery.

Based upon the results of this study, following a good performance, sport psychologists and/or practitioners should encourage recreational sport participants and athletes, at least when they do not have low self-esteem, to adopt an abstract processing style. In other words, when athletes perform well, they should be supported to think about the meanings, causes and implications of their performance because this may lead to a greater belief in the good outcome of future performances and may enhance their self-worth. This in turn may boost their motivation and, in the end, could contribute to enhanced performance in the long run.

### Conflict of Interest Statement

The authors declare that the research was conducted in the absence of any commercial or financial relationships that could be construed as a potential conflict of interest.

## Footnotes

1 Note that a study of Marigold et al. (2007) actually found that an abstract processing style induction for individuals with low self-esteem boosted positive feelings but had no effect in individuals with high self-esteem. However, the nature of their abstract manipulation was such that it implied that the compliment must have been significant and meaningful, thus leaving no room for such dampening cognitions or doubts. In fact, when the authors reworded the abstract manipulation in such a way that any potential doubts were possible, the effect disappeared.

2 When we deleted outliers, we found an effect of Condition, *t*(207) = –3.48, *p* < 0.01, β = –0.23, an effect of Self-Esteem, *t*(207) = 3.07, *p* < 0.01, β = 0.33, but there was no Condition × Self-Esteem interaction, *t* = –1.56.

3 When we deleted outliers, we found a marginal effect of Condition, *t*(207) = –1.83, *p* = 0.07, β = –0.11, an effect of Self-Esteem, *t*(207) = 4.05, *p* < 0.001, β = 0.35, and a Condition × Self-Esteem interaction, *t* = –2.31, *p* < 0.05, β = –0.20. Also, although the cronbach’s alpha of this scale was acceptable (α = 0.64), it was relatively low. Excluding item two of the scale resulted in an increased alpha of α = 0.81. Therefore, we ran the regression with three items as the total outcome score. For generalization to the self, we could not found an effect of Condition, *t*(214) = –1.42, *p* = 0.16, β = –0.09. But there was an effect of Self-Esteem, *t*(214) = 4.11, *p* < 0.001, β = 0.37, and a significant Condition × Self-Esteem interaction, *t*(214) = –2.28, *p* < 0.05, β = –0.21.
